# Antimony deposition onto Au(111) and insertion of Mg

**DOI:** 10.3762/bjnano.10.245

**Published:** 2019-12-18

**Authors:** Lingxing Zan, Da Xing, Abdelaziz Ali Abd-El-Latif, Helmut Baltruschat

**Affiliations:** 1 Institut für Physikalische und Theoretische Chemie, Universität Bonn, Römerstraße 164, D-53117 Bonn, Germany; 2 Key Laboratory of Chemical Reaction Engineering of Shaanxi Province, College of Chemistry & Chemical Engineering, Yan’an University, Yan’an, 716000 P.R. China; 3 Accumulator Materials Research (ECM), Zentrum für Sonnenenergie- und Wasserstoff-Forschung Baden-Württemberg (ZSW), Lise-Meitner-Str. 24, 89081 Ulm, Germany; 4 Permanent address: National Research Centre, Physical Chemistry Department, El-Bohouth St. Dokki, 12311 Cairo, Egypt

**Keywords:** alloy, antimony, Au(111), electrodeposition, insertion, STM

## Abstract

Magnesium-based secondary batteries have been regarded as a viable alternative to the immensely popular Li-ion systems owing to their high volumetric capacity. One of the largest challenges is the selection of Mg anode material since the insertion/extraction processes are kinetically slow because of the large ionic radius and high charge density of Mg^2+^ compared with Li^+^. In this work, we prepared very thin films of Sb by electrodeposition on a Au(111) substrate. Monolayer and multilayer deposition (up to 20 monolayers) were characterized by cyclic voltammetry (CV) and scanning tunneling microscopy (STM). Monolayer deposition results in a characteristic row structure; the monolayer is commensurate in one dimension, but not in the other. The row structure is to some extent maintained after deposition of further layers. After dissolution of the Sb multilayers the substrate is roughened on the atomic scale due to alloy formation, as demonstrated by CV and STM. Further multilayer deposition correspondingly leads to a rough deposit with protrusions of up to 3 nm. The cyclic voltammogram for Mg insertion/de-insertion from MgCl_2_/AlCl_3_/tetraglyme (MACC/TG) electrolyte into/from a Sb-modified electrode shows a positive shift (400 mV) of the onset potential of Mg deposition compared to that of a bare Au electrode. From the charge of the Mg deposition, we find that the ratio of Mg to Sb is 1:1, which is somewhat less than expected for the Mg_3_Sb_2_ alloy.

## Introduction

Rechargeable batteries have become essential energy storing devices, which are widely used in portable electronic devices and hybrid electric vehicles. Magnesium-based secondary batteries have been regarded as a viable “environmental friendly, non-toxic” alternative compared to Li-ion systems owing to their high volumetric capacity [1–4]. Unlike lithium, magnesium has no tendency to form dendrites during recharge [5]; but on the other hand, the Mg anode is covered with an insulation layer, which is different from the formation of a solid electrolyte interface (SEI) layer in Li systems. One of the main challenges in the commercialization of Mg-ion batteries is the incompatibility of the magnesium anode with the electrolytes because of the formation of this Mg^2+^ film.

Recently, Sb has been suggested as an alternative insertion material, because magnesium can form intermetallic compounds with antimony. In addition, Sb has a rhombohedral crystal structure, which can form an alloy over a wide composition range [6–7]. The high initial capacity of 298 mAh/g at 1C rate has been reported for electrochemical magnetization at electrodeposited Bi_0.88_Sb_0.12_ alloy by Arthur et al. [7]. However, the capacity declines to 215 mAh/g after 100 cycles with an electrolyte mixture of ethylmagnesium chloride, diethylaluminum chloride and anhydrous THF. A detailed, fundamental study of magnesium deposition/dissolution at a Sb-modified Au electrode surface has never been reported. Using this layer for such an insertion study in fundamental research offers the advantage of a better defined structure of the insertion compound as compared to the use of small particles in battery research.

The initial cyclic voltammetry study of antimony electrochemical deposition on a Au electrode was carried out by Jung [8], who found that antimony deposition on Au(100) and Au(111) in acid electrolyte undergoes two electrochemical processes involving an irreversible adsorption and underpotential deposition. This irreversible adsorption was attributed to oxygenous Sb(III) species, probably SbO^+^, which are formed in acid electrolyte and irreversibly adsorbed on the Au surface at a potential more positive than the underpotential deposition (UPD) potential [9]. Later, the fundamental research of this phenomenon of irreversible adsorption and UPD of Sb was investigated by electrochemical scanning tunneling microscopy (EC-STM). A detailed study on the structure of the irreversibly adsorbed oxygenous Sb(III) species and the Sb adlayer on Au(100) was carried out by Hara et al. and Yan et al. [9–10]. Jung investigated the structure of the irreversibly adsorbed oxygenous Sb(III) species on a Au(111) surface by an in situ STM [11] and Wu et al. [12] also investigated the electrodeposition of Sb on Au(111). It has been found that the formed adlayer has a 
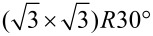
 structure on Au(111). The second process is the UPD process or the reduction of oxygenous Sb(III) species, which are from the bulk electrolyte. This was also investigated by other researchers [6–8,12], but the structure of underpotentially deposited Sb adlayer on Au(111) was not resolved on an atomic level. It is well known that Sb [13–15], Bi [16–18], etc. can be used to form bimetallic semiconductors. These semiconductors possess an interesting property and have been widely used in the field of electrocatalysis and materials.

It is therefore the aim of the present study to examine the initial stages of Sb-deposition on Au(111). Furthermore, we demonstrate the insertion of Mg into multilayers of Sb on Au(111) using a non-aqueous electrolyte, such as MgCl_2_/AlCl_3_/tetraethylene glycol dimethyl ether (tetraglyme) (MACC/TG). The reversibility of insertion/de-insertion is demonstrated, and thus the suitability of such adlayers for studying the fundamentals of the insertion reaction is demonstrated. In a follow up paper, we will study the kinetics of the insertion process in more detail [19].

## Experimental

### Chemicals, materials and electrolyte

**Figure 1:**
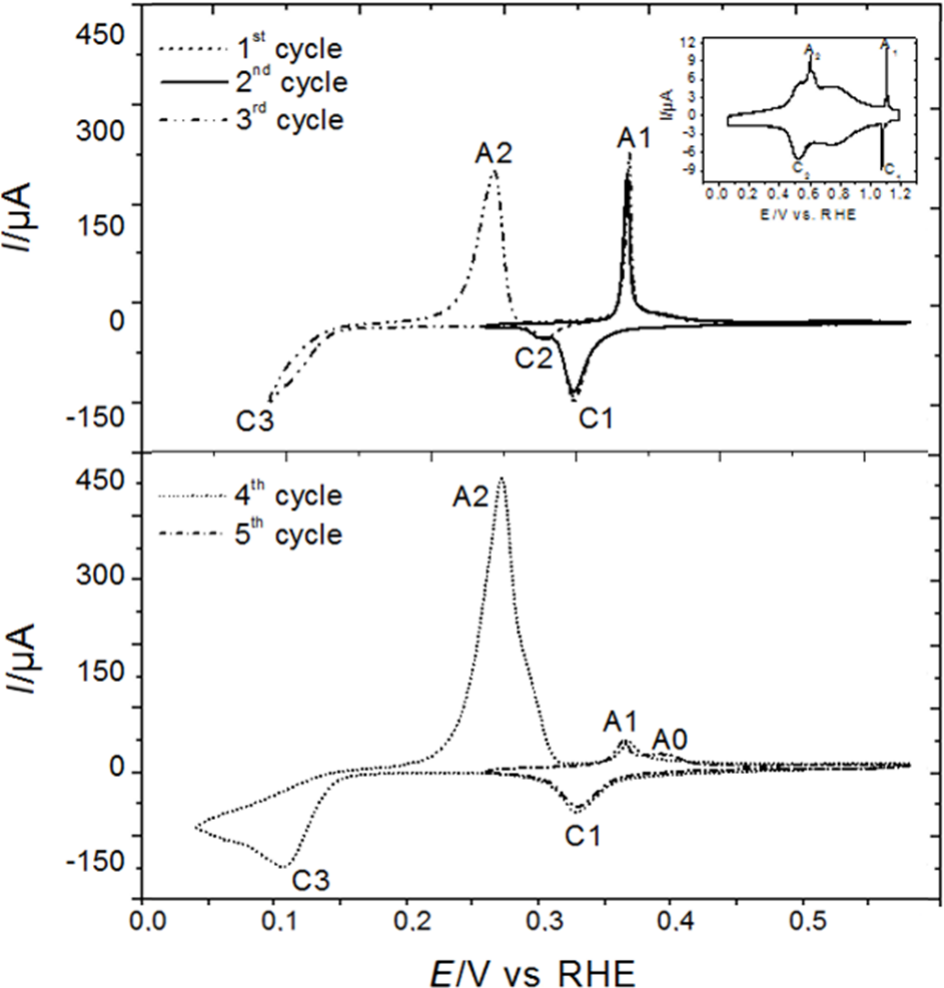
Cyclic voltammograms of Sb deposition on Au(111) in 0.25 mM Sb_2_O_3_/0.5 M H_2_SO_4_ electrolyte saturated with Ar in an H-cell at 10 mV s^−1^. Inset: Cyclic voltammogram of Au(111) in 0.1 M H_2_SO_4_ solution saturated with Ar in an H-cell at 50 mV s^−1^ to demonstrate the Au(111) surface.

The Au(111) single crystal was prepared by cooling down after flame annealing in pure argon (Air Liquid, 99.999%,) atmosphere as described elsewhere [20]. A typical cyclic voltammogram of Au(111) in 0.1 M H_2_SO_4_ is shown in the inset of Figure 1. The interpretation of the voltammetric feature of Au(111) has been previously reported [21–23]. All aqueous electrolytes were prepared using 18.2 MΩ Milli-Q water and deaerated with high purity argon gas for at least 15 min before use. The electrochemical deposition of antimony at the Au electrode was done in 0.25 mM Sb_2_O_3_ (99.999%, Aldrich) and 0.5 M H_2_SO_4_ electrolyte.

A Au(111) electrode and an antimony-modified Au(111) electrode were used as working electrodes for Mg deposition measurements. Magnesium foil was used as a counter electrode and another one as a reference electrode. All the magnesium electrochemical deposition measurements were carried out in a MBraun glovebox (H_2_O < 0.5 ppm, O_2_ < 0.5 ppm) in a similar manner as described in [19].

### Cyclic voltammetry (CV)

A polycrystalline Au electrode with a similar size was also employed. All aqueous electrolytes were prepared by 18.2 MΩ Milli-Q water and deaerated with high purity argon gas for at least 15 min before use. Electrochemical measurements in 0.1 M H_2_SO_4_ (spectro pure grade) were carried out in a conventional three electrode glass H-cell consisting of three compartments for fixing the working electrode, reference electrode and counter electrode. The working electrode is placed in the central compartment and contacted with solution in a hanging meniscus configuration. The reference electrode is placed in the compartment where it is connected to the central compartment with a Luggin capillary. The counter electrode is placed in the compartment where it can be separated from the central compartment by a glass frit.

All electrochemical measurements were carried out using a bi-potentiostat purchased from Pine Instruments, Inc. (model AFBPC1) in combination with LabVIEW software (National Instruments GmbH, Munich, Germany) for recording the cyclic voltammograms (CVs).

### Electrochemical scanning tunneling microscopy (EC-STM) measurements

All EC-STM measurements were performed with an Agilent Technologies 5500 scanning probe microscope (SPM) and a commercially available STM scanner (Molecular Imaging/Agilent Technologies) fitted with a so-called STM/AFM electrochemical cell as previously described [24]. Pt and Au wires were used as a quasi-reference electrode (*E*_Pt/PtO_ = 0.9 V vs RHE) and a counter electrode, respectively. The reference electrode was immersed in a small compartment filled with the same electrolyte and separated from main compartment by a capillary. Pt/Ir (90:10) STM tips with a diameter of 0.25 cm were prepared by etching in a 2 M KOH/4 M KSCN bath and coated with hot-melt glue containing different types of polymer (provided by Steinel) to minimize faradaic current. All of the EC-STM measurements were performed in a glass chamber purged with argon at room temperature.

### Preparation of MACC/TG

All chemicals were purchased from Sigma-Aldrich. Tetraglyme was distilled over sodium and stored over molecular sieves (3 Å) until the water content was less than 5 ppm. The water content was determined by coulometric Karl Fischer titration (Mettler Toledo). MgCl_2_ was heated overnight under vacuum at 290 °C and then stored under thionyl chloride for 1 week. At low pressure, the thionyl chloride was removed completely. All materials were handled in an Argon-filled glovebox. The MACC electrolyte was prepared by adding tetraglyme (20.5 mL) to MgCl_2_ (0.966g). While stirring, the AlCl_3_ (1.368 g) was added stepwise. The whole mixture was then stirred overnight after addition of an equivalent amount of MgH_2_ to reduce the water content (analogous as described in [19]).

## Results and Discussion

### Electrochemistry of antimony on Au(111)

The cyclic voltammograms of Sb deposition at underpotential and overpotential on Au(111) in 0.5 M H_2_SO_4_ containing 0.25 mM Sb_2_O_3_ electrolyte saturated by argon are shown in Figure 1. Two peaks were observed in the underpotential deposition region. The first cathodic peak C1 (≈+0.3 V) is due to the reduction of preadsorbed oxygenous Sb(III) species (SbO^+^). In a highly acidic electrolyte (0 < pH < 1), the main species of antimony is SbO^+^ as reported by Wu et al. [12]. The following small peak C2 (≈+0.28 V) is due to the reduction of oxygenous Sb(III) species from bulk solution.

**Figure 2:**
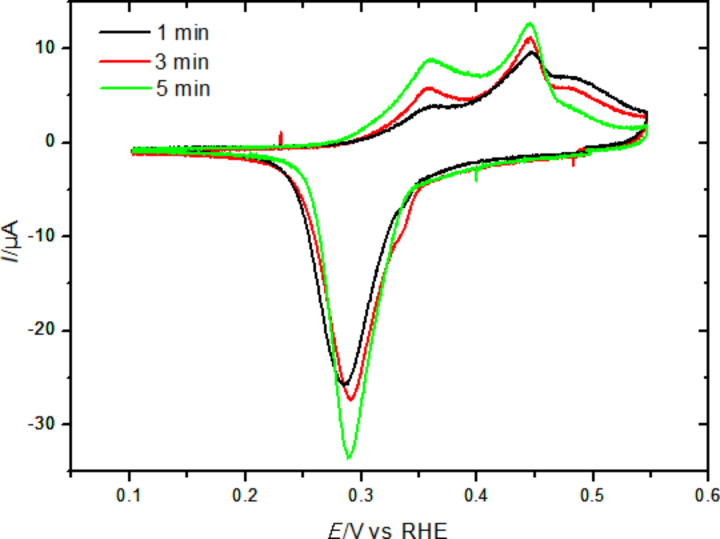
Cyclic voltammograms of Sb species on Au(111) in 0.1 M H_2_SO_4_ electrolyte saturated with Ar at the sweep rate of 10 mV s^−1^. The irreversible adsorption of Sb species on Au(111) was induced after the electrode surface was in contact with Sb-containing electrolyte in the hanging meniscus configuration for 1, 3 and 5 min at open circuit potential (≈0.6 V). A nearly complete desorption of Sb from the Au(111) surface could only be achieved after extensive cycling in pure 0.1 M H_2_SO_4_. Then, the sulfate adsorption peaks and the corresponding spike at 1.05 V becomes discernible (see Supporting Information File 1, Figure S1). Obviously, some Sb remains incorporated in the Au surface and the Au(111) surface does not recover complete smoothness.

The total charge density of peaks C1 and C2 (≈320 µC cm^−2^) suggests that the coverage of a monolayer is around 0.44 by assuming a one to one ratio of Sb to Au atoms for a hypothetical monolayer (and a 3e^−^ process), which is close to the reported value of 0.43 [8]. However, Itaya et al. [9] have investigated the Sb structure at Au(100) and they found the total charge due to Sb UPD and irreversible adsorption corresponding to the coverage of 0.63, which could be due to the influence of anions because of the co-adsorption. The charge of the corresponding dissolution peak A1 at +0.34 V amounts to 290 µC cm^−2^. The charge ratio of the anodic peak A1 to the cathodic peaks C1 and C2 in the first potential cycle for Sb stripping/deposition in UPD region gives a value of 90%. This residual 10% (30 µC cm^−2^) may indicate some alloy formation from which Sb cannot again be deoxidized in the potential range and time scale of the experiment. Figure 2 shows the cyclic voltammograms of Sb species on Au(111) electrodes which was induced by immersing the electrode surface into the Sb-containing electrolyte for 1, 3 and 5 min at open circuit potential in 0.1 M H_2_SO_4_ electrolyte. The electrode was rinsed with plenty of 0.1 M H_2_SO_4_ electrolyte before recording the CVs. The reduction and oxidation of Sb species on Au(111) were observed, suggesting the irreversible adsorption of Sb species (probably SbO^+^) on the Au(111) the surface upon contact with the Sb-containing electrolyte at open circuit potential. These the cyclic voltammograms are similar and the charge of the cathodic peak in the first cycle in these three cases (contact for 1, 3 and 5 min) is around 210 µC cm^−2^, indicating that the irreversible adsorption process is fast and the coverage of the irreversibly adsorbed Sb species on Au(111) is around 0.30 (assuming a 3e^−^ transfer), which is in good agreement with the value obtained in the literature [11]. It was also observed that the prolonged contact time (≥1 min) did not effect on the CV on Au(100) electrode surface [9].

The reduction peak potential is identical to the first reduction peak C1 in Figure 1. In agreement with the literature, we can therefore conclude that peak C1 in Figure 1 also corresponds to the reduction of the irreversibly adsorbed Sb species and peak C2 to further UPD of Sb. The peak C1 observed by Wu et al. [12] is smaller than that observed by us. We also observed a smaller peak C1 on a polycrystalline Au electrode. Therefore, this difference is probably due to the roughness of the Au(111) surface, resulting from repeated alloying and dealloying during the cycling to obtain stable voltammetry in [12]. Upon extension of the potential cycle to more negative potentials, bulk Sb starts around 0.13 V and gives rise to the peak C3 and the corresponding dissolution peak A3. Peak C1 and peak A2 largely decrease after extensive bulk deposition of nearly two monolayers in the fourth cycle. We attribute the decrease of peak A2 and C1 to the formation of a Au–Sb alloy in the bulk deposition region, A AuSb_2_ surface alloy has been previously reported by Stegemann et al. [25] after deposition of Sb in UHV. Less Sb-UPD can form on Au–Sb alloy than on Au. In addition, less adsorbed Sb-species (SbO^+^) is adsorbed at positive potential, therefore the corresponding redox process becomes less prevenient. The presence of an anodic peak (A1) is then due to the stripping of Sb from the alloy.

It is interesting to note that the difference between peaks C1 and C2 (now 146 µC cm^−2^) to that of A1 and A2 (now 100 µC cm^−2^) is 40 µC cm^−2^, which is about the same as in the first cycle and probably corresponds to same continuous incorporation of Sb into the surface to form the surface alloy.

The behavior of Sb UPD on Au(111) is somewhat different from that on Pt(111) [26]. There, after the first deposition of Sb in peak C1 (occurring at 0.4 V vs RHE), a roughening transition occurs and neither dissolution nor further deposition of Sb occurs. However, here in the case of Au(111), such an incorporation and decrease of the peak C1 occurs only after extension of the sweep to more negative potentials.

### Electrochemical scanning tunneling microscopy (EC-STM) measurements on Sb/Au(111)

**Figure 3:**
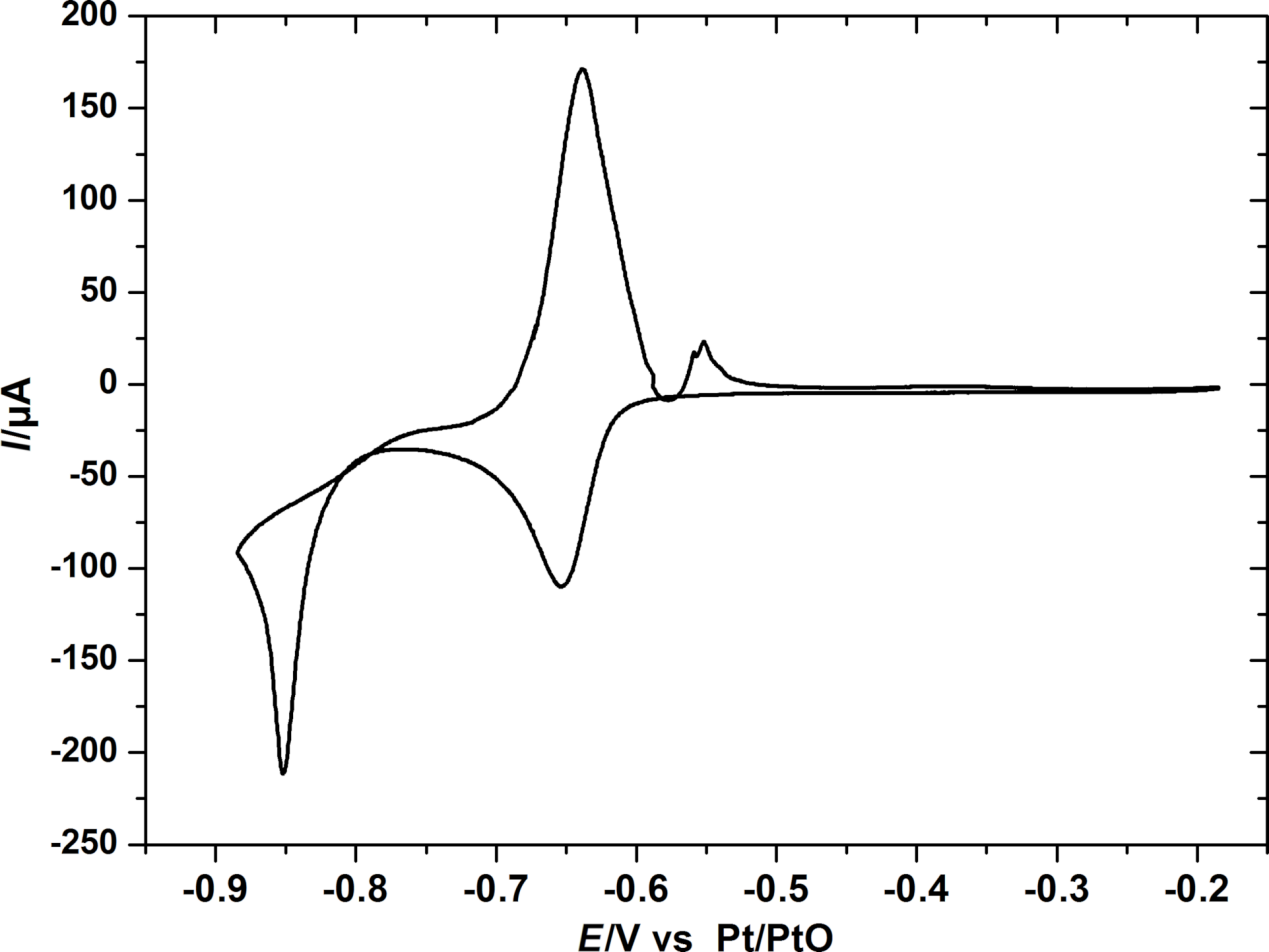
Cyclic voltammogram of Sb deposition on Au(111) in 0.25 mM Sb_2_O_3_/0.5 M H_2_SO_4_ electrolyte in the STM cell at 10 mV s^−1^.

A similar cyclic voltammogram of Sb deposition on Au(111) in 0.25 mM Sb_2_O_3_/0.5 M H_2_SO_4_ electrolyte was obtained in the STM cell during the STM measurements as shown in Figure 3. It is similar to that recorded in the classical glass cell when the potential is converted to versus reversible hydrogen electrode (RHE) using *E*_(Pt/PtO)_ ≈ 0.9 V vs RHE. The anodic monolayer oxidation peak is much smaller than the cathodic one, because of the extension of the sweep into the bulk deposition regions. However, the larger negative current on the negative side of the UPD peak and some superimposed negative current may be due to oxygen reduction, which starts at ≈0.36 V vs RHE (or −0.54 V vs Pt/PtO) on the Au electrode in 0.5 M H_2_SO_4_ [27]. The bulk deposition peak is much sharper because of the different diffusion behaviors in the small volume STM cell, which somewhat resembles that of thin layer cells [28]. A further difference is that both anodic processes are shifted in positive direction by 50 mV, which might be due to some instability of the pseudo-reference electrode.

**Figure 4:**
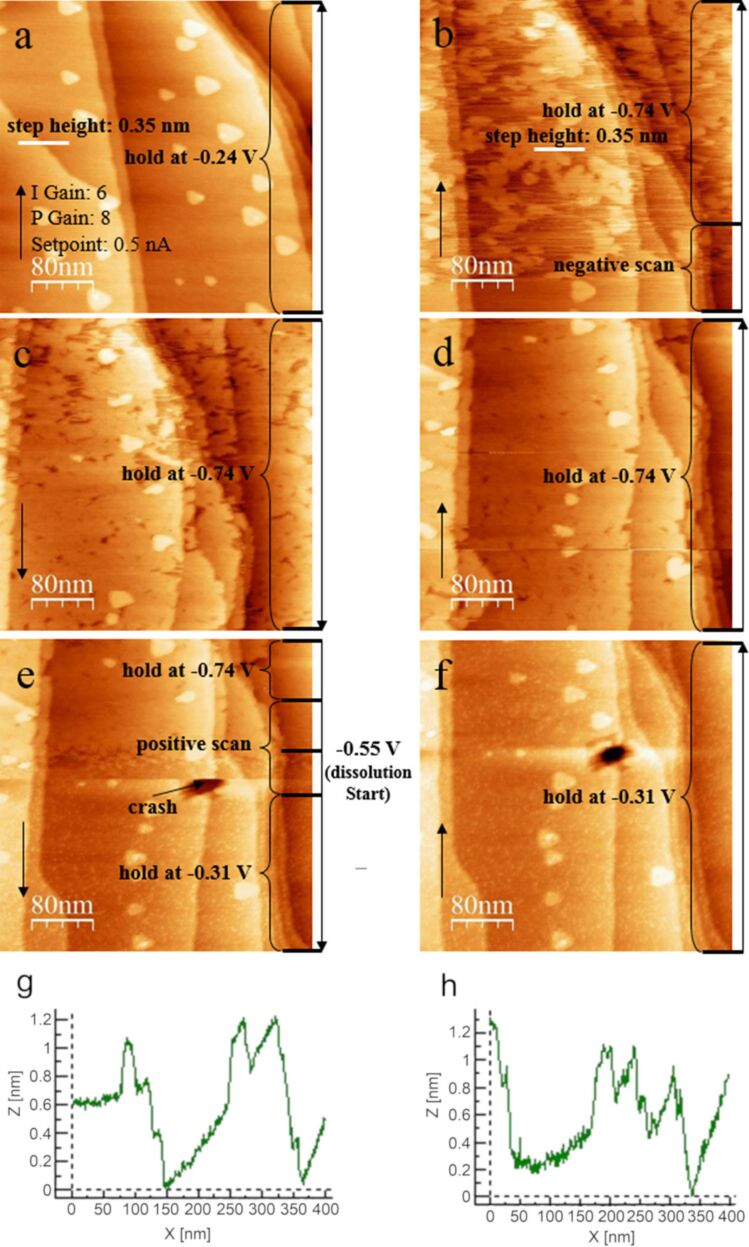
Sequential STM topographic images of Sb deposition/dissolution on Au(111) in 0.25 mM Sb_2_O_3_/0.5 M H_2_SO_4_ electrolyte. (a) Initial Au(111) surface at open circuit potential of −0.24 V. (b) The electrode potential was scanned from −0.24 to −0.74 V and then stopped at −0.74 V, the height of Sb adlayer is shown with cross section in the image g. (c, d) The electrode potential was held at −0.74 V, followed by formation of a complete monolayer. (e) The electrode potential was scanned back from −0.74 to −0.31 V and then stopped at −0.31 V, followed by dissolution of the monolayer. (f) The electrode potential was held at −0.31 V. Line-by-line correction was applied for the topographic images. The two profiles (g, h) correspond to the two black lines in image a and f. Sample bias of 50 mV, set point = 0.5 nA and scan rate of 3.04 ln/s. Integral gain: 6, proportional gain: 8. Arrows indicate the scan direction.

An in situ observation of Sb underpotential deposition/dissolution on/from the Au(111) surface in 0.5 M H_2_SO_4_ containing 0.25 mM Sb_2_O_3_ electrolyte in the STM cell is shown in Figure 4. In all EC-STM measurements, a Pt quasi-reference electrode (Pt/PtO) electrode was used as the reference electrode and the potential was scanned from −0.24 V (open circuit potential) to −0.74 V, which is in the potential range of UPD. At open circuit potential, the Au(111) surface is decorated by tiny particles and some small islands are observed as shown in Figure 4a. These small Au islands are formed when the reconstruction of the Au(111) surface is lifted at the rest potential in solution. When the potential was scanned negatively, the nucleation on the terraces and epitaxial 2D growth started at ≈0.6 V (see Figure 4b). The Sb monolayer islands were formed with the same height as the step height of the freshly prepared Au(111) which is 0.2 nm (see Figure 4a). With the continuous growth at the potential of −0.74 V, a complete monolayer was formed within approximately 3.5 min as shown in Figure 4c,d. However, this monolayer can be dissolved quickly when the potential is scanned positively to −0.31 V (see Figure 4e). However, some tiny particles appeared on the surface after dissolution as shown in Figure 4f, suggesting that the deposited Sb species cannot be dissolved completely and a Au–Sb alloy was formed. This confirms the result obtained in the electrochemical measurement.

**Figure 5:**
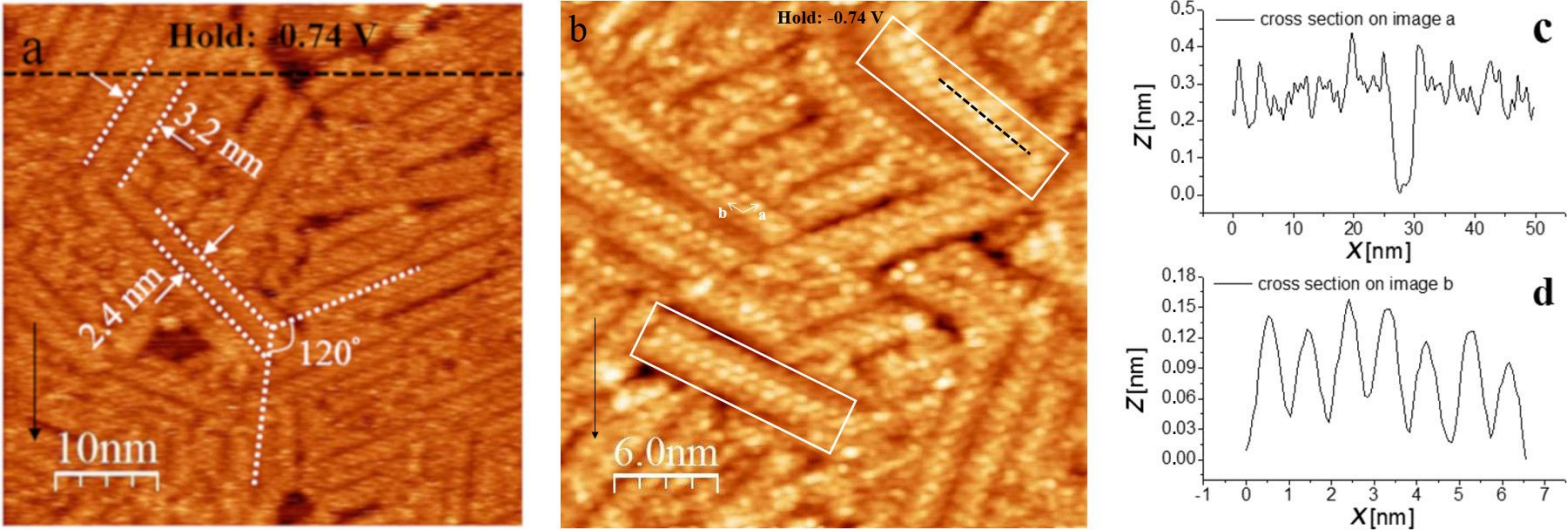
STM images of the Sb adlayer structure on Au(111) in 0.25 mM Sb_2_O_3_/0.5 M H_2_SO_4_ electrolyte at −0.74 V. (a) 50 × 50 nm; (b) 30 × 30 nm; (c) the cross section of image a; (d) the cross section of image b. Sample bias of 50 mV, set point = 0.5 nA and scan rate of 12 ln/s. Integral gain: 2 and proportional gain: 3. Arrows indicate the scan direction. Line-by-line correction is used for images.

The atomic resolution of the Sb adlayer structure on the Au(111) surface was obtained at the potential of −0.74 V and is shown in Figure 5. As shown in Figure 5a, the distance between the rows is typically 2.4 to 3.2 nm. In different domains, their direction varies by 60°. However, in some cases also angles of approximately 20° were observed. The distance between two adjacent Sb atoms along the black dotted line in image b is shown in Figure 5d. To get accurate lattice parameters, the error induced by the thermal drift was eliminated by drift calibration and the Sb adlayer lattice vectors were corrected by using the calibration matrix from the known adlayer lattices of sulfate on Au(111), as outlined in detail by Iqbal et al. [24]. The orientation of the substrate was kept almost the same (±10°) for each experiment. The corrected distances between the two adjacent maxima of visible atoms along vector 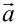 and vector 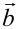 (see Figure 5b) were found to be about 1 nm for 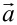 and (more precisely) 0.82 nm for 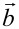, respectively. Both vectors include an angle of 114.6°, i.e., close to 120°. This spacing is considerably larger than that of the initial Sb adlayer which is formed by the reduction of the adsorbed oxygenous Sb species with the structure of 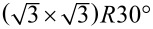. From the area between the vectors 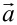 and 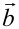 we estimate a packing density of the maxima of around 10%, which is much less than that of the 
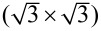
 structure (theoretically 33%, Supporting Information File 1, Figure S2) and that calculated from the deposition charge (44%, Figure 1). Roughly, only every fourth atom is visible in the STM image, or every second in one dimension. Unfortunately, because of the incommensurability in the direction other than the row, no structure model can be given.

The angle between the direction of the rows and the [110] direction of the substrate (that of the dense atomic rows) is very roughly 13°. Therefore we very tentatively assume for the vector in the direction of the rows 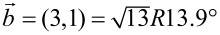 with a theoretical length of 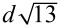 = 1.04 nm (distance between Au atoms *d* = 0.282 nm) involving one atom in an on top position and another one in a bridge position (not visible). However, it is more likely that the configuration is one atom in a three field hollow site position (not visible) and a further one in a near on top position (visible).

Wu et al. also observed a similar row structure of Sb on Au(111) [12], but it is not as clear as pure data. A somewhat similar row structure of Sb has been observed on Au(100) in perchloric acid electrolyte by Hara et al. [9].

**Figure 6:**
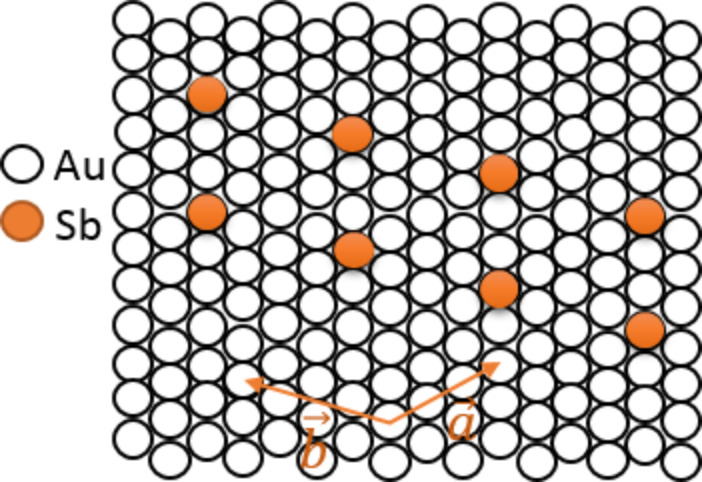
Tentative model of the Sb adlayer structure on Au(111) in the direction of the rows (direction of vector 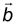 = (3,1), cf. Figure 5b)). White circles: Au atoms; yellow circles: Sb atoms. Only atoms that are “visible” by STM are shown, as discussed in the text, and the number of adsorbed Sb atoms is probably four times higher. A second row is also indicated assuming approximately a vector of 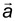 = 0.4, where its position with repect to the first one is speculative and only approximate.

Unfortunately, the resolution between the rows is not sufficient to estimate a second (incommensurate) lattice vector 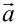 of the adsorbate. We therefore only show a model for the arrangement of the atoms in the direction of the rows in Figure 6 (vector 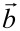).

### Bulk deposition of Sb on Au(111)

**Figure 7:**
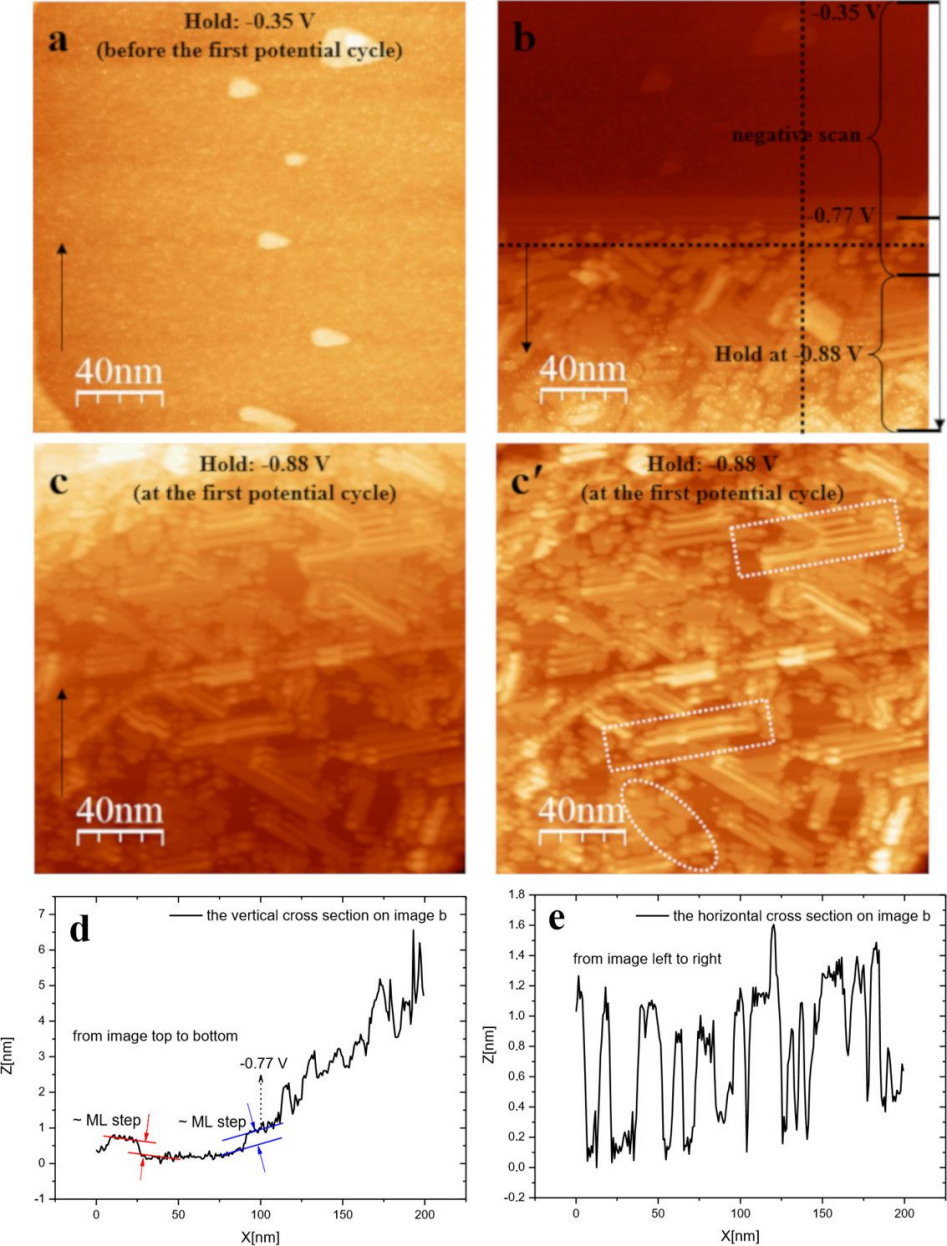
STM images of Sb overpotential deposition on Au(111) during the first potential cycle in 0.25 mM Sb_2_O_3_/0.5 M H_2_SO_4_ electrolyte. (a) Initial Au(111) surface at the potential of −0.35 V. (b) The electrode potential was scanned negatively from −0.35 to −0.88 V and then stopped at −0.88 V. (c, c') The electrode potential was held at −0.88 V. (d) The cross section of image b (black vertical dotted line). (e) The cross section of image b (black horizontal dotted line). Sample bias = 50 mV, set point = 0.5 nA and scan rate = 3.04 ln/s. Integral gain: 6 and proportional gain: 8. The arrows indicate the scan direction. Plane correction was used for images a, b and c. Line-by-line correction was used for image c'.

A freshly prepared Au(111) surface was employed for observing the bulk adlayer structure of Sb formed on its surface. The bulk deposition process is demonstrated in Figure 7. The Au(111) surface at the potential of −0.35 V in the Sb^3+^-containing electrolyte is shown in Figure 7a. As the potential was scanned negatively from −0.35 to −0.88 V (see Figure 7b, c and c'), the bulk deposition of Sb starts at ≈−0.8 V and both regular and irregular three dimensional (3D) structures are observed: row structures and island-like structures. The increase in the height of the Sb deposits with the initial decrease of the potential and then with the holding the potential at −0.88 V is shown in Figure 7d. The horizontal cross section on Figure 7b is shown in Figure 7e. It shows that the ≈1 nm height of the Sb deposits was formed at that time. At the bottom of Figure 7b, the height of the Sb deposits reaches to ≈6 nm, which is around 30 layers. A somewhat atypical Stranski–Krastanov growth was thus observed during overpotential deposition.

**Figure 8:**
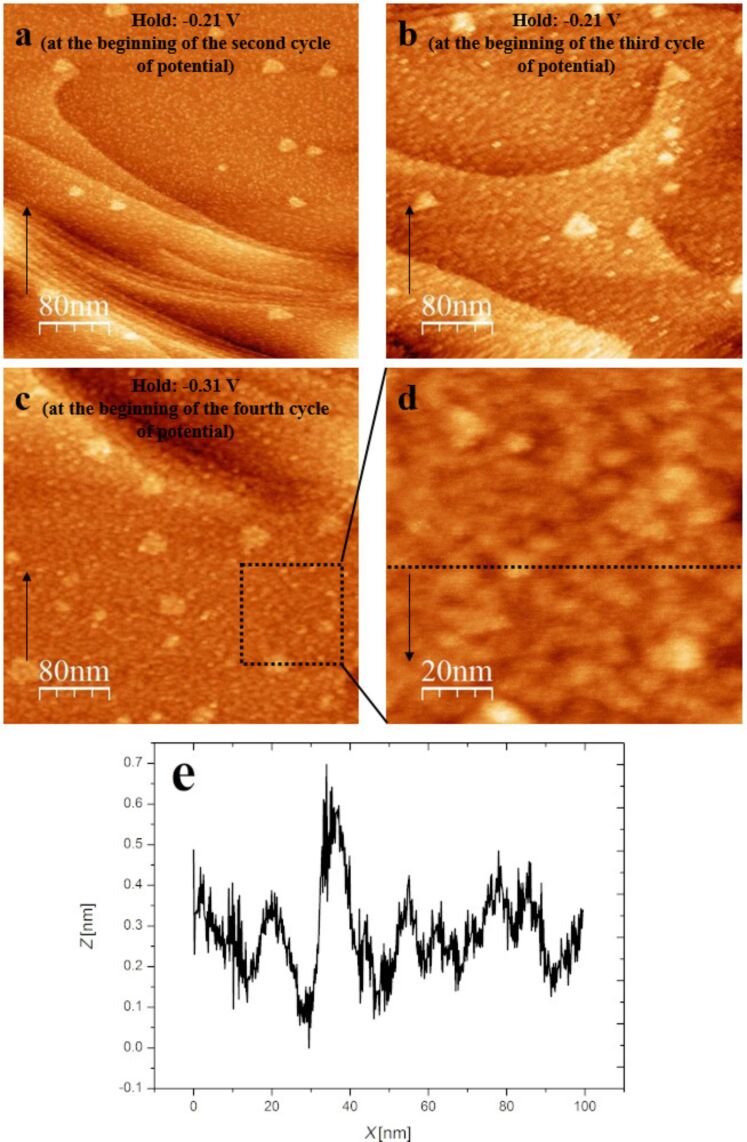
STM images of the Au(111) surface after Sb stripping in 0.25 mM Sb_2_O_3_/0.5 M H_2_SO_4_ electrolyte. (a) The potential was held at −0.21 V after the first potential cycle. (b) The potential was held at −0.21 V after the second potential cycle. (c) The potential was held at −0.31 V after the third potential cycle. (d) The zoomed in details of image c (black box) and (e) the cross section of image d (black dotted line). Sample bias = 50 mV, set point = 0.5 nA and scan rate = 3.04 ln/s. Integral gain: 6 and proportional gain: 8. The arrows indicate the scan direction. Line-by-line correction was used for the images.

After stripping of Sb at −0.21 V the Au(111) substrate is not smooth again. Figure 8a shows the Au(111) surface after stripping of Sb at −0.21 demonstrating a severe roughness on the atomic scale. As shown in Figure 8b and c, the resulting Au(111) surface becomes more and more rough after each further deposition and dissolution process, which is an indication of the alloy formation [10,25,29]. The root mean square of the roughness (RMS roughness) of the terraces were determined to be 0.088, 0.095 and 0.130 on the image a, b and c, respectively.

**Figure 9:**
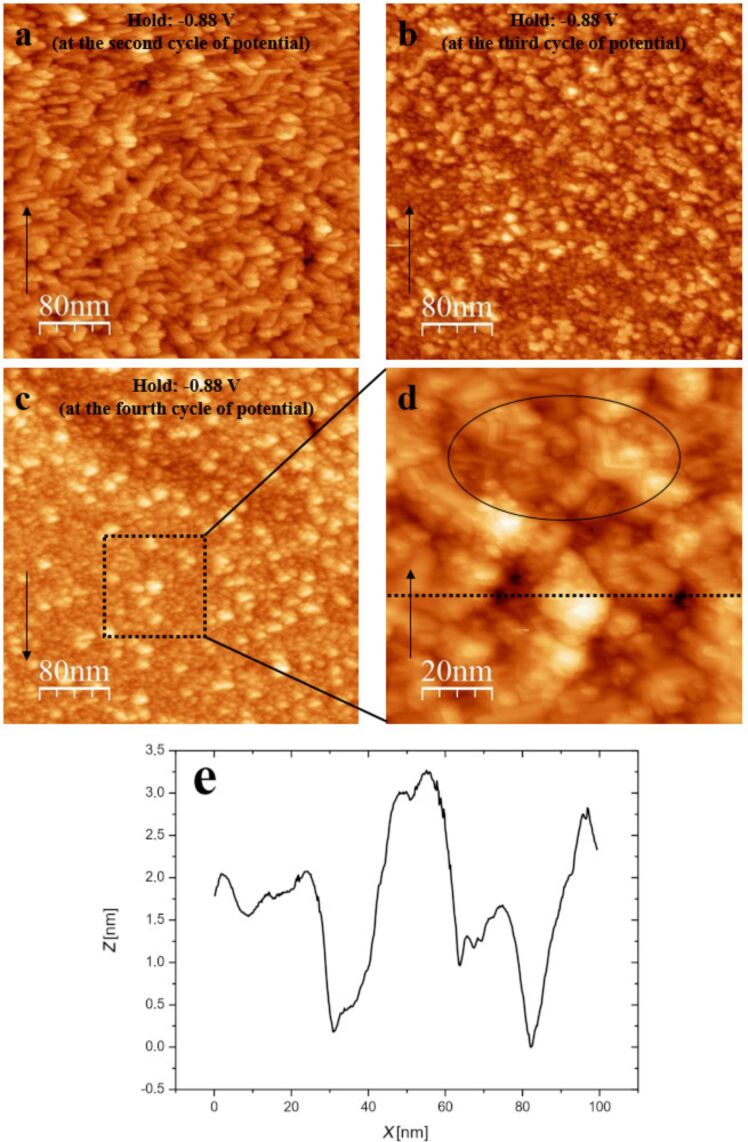
STM images of Sb overpotential deposition on Au(111) in 0.25 mM Sb_2_O_3_/0.5 M H_2_SO_4_ electrolyte. (a) The potential was held at −0.88 V at the second potential cycle. (b) The potential was held at −0.88 V at the third potential cycle. (c) The potential was held at −0.88 V at the fourth potential cycle. (d) The zoomed in details of image c (black box) and (e) the cross section of image c (black dotted line). Sample bias = 50 mV, set point = 0.5 nA and scan rate = 3.04 ln/s. Integral gain: 6 and proportional gain: 8. The arrows indicate the scan direction. Line-by-line correction is used for the images.

Subsequent deposition of Sb at −0.88 V during potential holds within following potential cycles are shown in Figure 9. The deposited Sb were also formed in a row structure during the second potential cycle (see Figure 9a). However, most of the deposited Sb formed a particle-like structure during the third and fourth potential cycle (see Figure 9b and c), but some row structure could still be observed (see Figure 9d).

### Mg insertion and deposition

**Figure 10:**
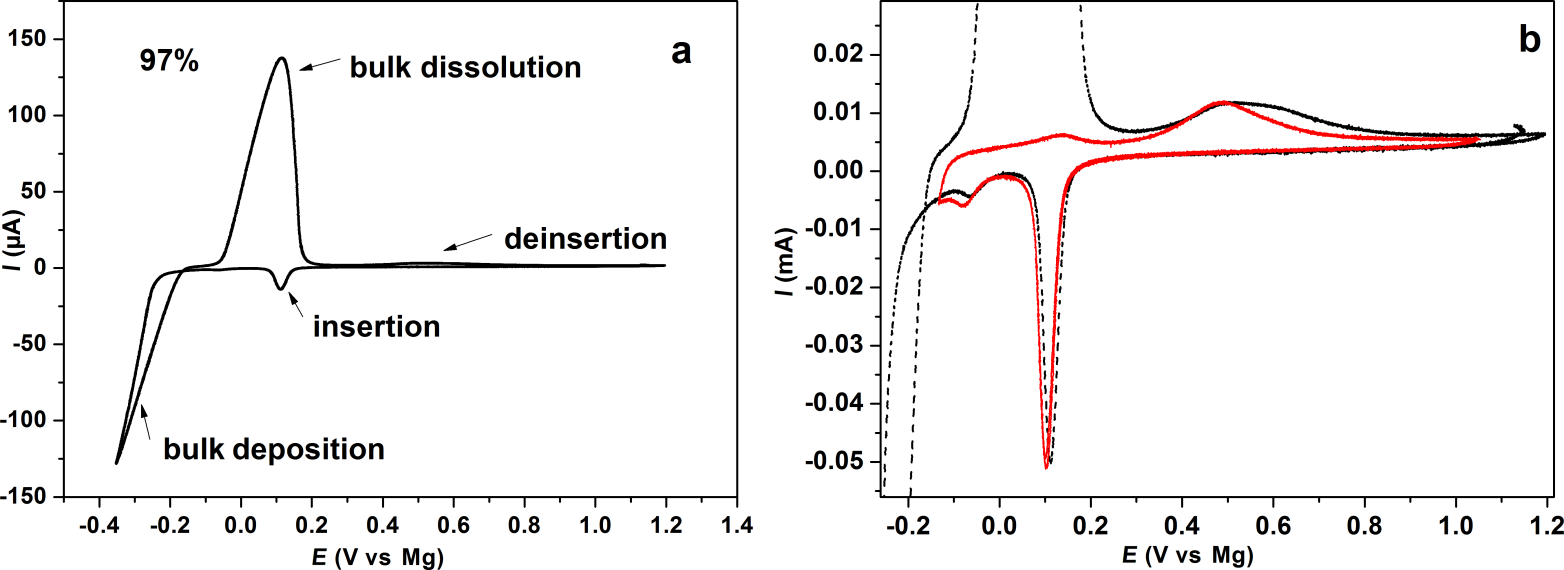
(a) Cyclic voltammograms of Mg deposition/dissolution and Mg insertion/de-insertion at the single Au electrode (Au(111)) in MACC/tetraglyme at a sweep rate of 1 mV s^−1^. (b) Details of Mg insertion/de-insertion at the Sb-modified electrode in the potential range of −0.1 V to 1.1 V.

In some preliminary further experiments we examined the suitability of this thin Sb-layer for the study of intercalation of Mg. Figure 10 shows the electrochemical deposition and stripping and insertion/de-insertion behavior of Mg into the Sb-adlayer at the Au(111) electrode in 0.5 M MgCl_2_/0.5 M AlCl_3_ in tetraglyme. The electrochemical deposition and stripping behavior of Mg at the single crystalline gold electrode is similar to the behavior of Mg at a polycrystalline gold electrode [19]. The small peak at 0.1 V (vs Mg) is due to the insertion of Mg into the Sb-adlayer and is not visible in the absence of Sb. The bulk deposition of Mg starts below −0.2 V. During the anodic going sweep, the current is still negative in the potential range of −0.35 to −0.2 V due to the continuous deposition of Mg. At *E* > 0.2 V, Mg is dissolved. The red curve in Figure 10 is limited to the potential range positive of Mg bulk deposition. Therefore, this shows only the insertion/de-insertion behavior. The two observed peaks are the same as in the black curve, which indicates the insertion and de-insertion of Mg without Mg-bulk deposition. The ratio between the faradaic charge of anodic and cathodic gives the apparent coulombic efficiency. Therefore, 99.8% of the inserted magnesium is dissolved in the subsequent anodic sweep. The reversibility of the magnesium bulk deposition/dissolution is nearly 97%. From the Sb deposition charge (10.36 mC cm^−2^), the amount of the deposited Sb was calculated to be 20 monolayers, which is comparable to the STM results. The amount of the inserted Mg was 6.39 mC cm^−2^. The ratio between the amounts of the inserted Mg (2e^−^) to the deposited Sb (3e^−^) was thus found to be 1:1, which is less than the theoretical value 3:2 (Mg_3_Sb_2_).

We attribute this to the insertion of Mg into the Sb-deposited layers and formation of magnesiated binary phase of antimony (Mg_3_Sb_2_), according to the following equation:

[1]
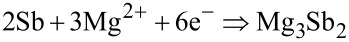


where the onset potential is 400 mV more positive than that of bulk deposition at the bare Au electrode (Figure 10a and Supporting Information File 1, Figure S3). From the thermodynamic data for Mg_3_Sb_2_ alloy formation [30], 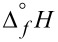 = −300193.567 J/mol, and 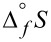 = −52.50 J/mol·K, the calculated potential for Mg–Sb alloy formation at room temperature is ≈550 mV (vs Mg). Therefore, the experimental positive shift in the overpotential of Mg deposition during the insertion of Mg into Sb adlayers is in agreement with the theoretical value. After the saturation of the host layers, bulk deposition of Mg started with the corresponding increase of the cathodic current.

The discrepancy between the theoretical stoichiometry and the experimental deposition change ratios is explained by the thin Sb-layer, which does not allow a complete conversion to the bulk Mg_3_Sb_2_ phase. In a forthcoming study, for somewhat thicker Sb layers on poly crystalline Au, we will show that the ratio of deposition charge for Mg and Sb is indeed 3:2 [19]. In this forthcoming study, we will also give further evidence for the alloy formation and present a study on the rate of this process.

## Conclusion

The electrochemical deposition of antimony on Au(111) was investigated in 0.5 M H_2_SO_4_ containing 0.25 mM Sb_2_O_3_ by cyclic voltammetry and electrochemical scanning tunneling microscopy (EC-STM). Two peaks were observed in the UPD region, one at 0.3 V due to the reduction of the irreversibly adsorbed oxygenous Sb(III) species and the other one at 0.28 V due to the reduction of oxygenous Sb(III) species from bulk electrolyte. The coverage of the Sb monolayer was calculated to be 0.44 whereas the irreversible adsorption accounted for ≈0.3. By observation with STM, Sb nucleation prefers to start at the active sites on the terrace, and then epitaxial 2D growth occurs to form the row structure with the width of ≈2.4 or ≈3.2 nm and the height of 0.35 nm. The angle between the two different oriented domains is around 120°, which suggests that they are probably aligned along the densely packed (111) rows of the substrate. Some vacancies appeared between two parallel rows. The corrected distances between two Sb neighboring atoms were calculated to be 0.851 nm and 0.856 nm, respectively. The coverage of the Sb UPD adlayer obtained from the STM results is in good agreement with the CV result. Furthermore, the bulk adlayer structure is dependent on the roughness of the surface, which increased with the continuous deposition and dissolution of antimony in this case due to alloy formation between Sb and Au. Therefore, a structure change of the bulk adlayer from rows to a particle-like structure was observed during the continuous potential cycles.

Magnesium deposition/dissolution on Au(111) with Sb-modified electrodes was investigated in MACC. Interestingly, at the Sb-modified Au electrode, a cathodic peak appears at 400 mV more positive than the onset potential of bulk deposition at the Au electrode. We propose that this potential shift is due to the formation of Mg_3_Sb_2_ alloy during the insertion of magnesium into Sb adlayers.

## Supporting Information

File 1 Additional experimental details.

## References

[1] Muldoon J, Bucur C B, Gregory T (2014). Chem Rev.

[2] Ma Z, MacFarlane D R, Kar M (2019). Batteries Supercaps.

[3] Luo J, Bi Y, Zhang L, Zhang X, Liu T L (2019). Angew Chem, Int Ed.

[4] Dong H, Liang Y, Tutusaus O, Mohtadi R, Zhang Y, Hao F, Yao Y (2019). Joule.

[5] Aurbach D, Cohen Y, Moshkovich M (2001). Electrochem Solid-State Lett.

[6] Cheng Y, Shao Y, Parent L R, Sushko M L, Li G, Sushko P V, Browning N D, Wang C, Liu J (2015). Adv Mater (Weinheim, Ger).

[7] Arthur T S, Singh N, Matsui M (2012). Electrochem Commun.

[8] Jung G, Rhee C K (1997). J Electroanal Chem.

[9] Hara M, Inukai J, Yoshimoto S, Itaya K (2004). J Phys Chem B.

[10] Yan J W, Wu Q, Shang W H, Mao B W (2004). Electrochem Commun.

[11] Jung C, Rhee C K (2004). J Electroanal Chem.

[12] Wu Q, Shang W-H, Yan J-W, Mao B-W (2003). J Mol Catal A: Chem.

[13] Butterman W C, Carlin J F (2004). Open-File Rep - U S Geol Surv.

[14] Xu D, Shen S, Zhang Y, Gu H, Wang Q (2013). Inorg Chem.

[15] Ipser H, Flandorfer H, Luef C, Schmetterer C, Saeed U (2007). J Mater Sci.

[16] Jüttner K (1986). Electrochim Acta.

[17] Kokkinidis G (1986). J Electroanal Chem Interfacial Electrochem.

[18] Jeffrey C A, Harrington D A, Morin S (2002). Surf Sci.

[R19] 19Xing, D.; Latif, A. A. A.; Zan, L. X.; Baltruschat, H. Unpublished work, 2019.

[20] Hernandez F, Sanabria-Chinchilla J, Soriaga M P, Baltruschat H, Birss V I, Josowicz M, Evans D (2004). Electrode Processes VII.

[21] Angerstein-Kozlowska H, Conway B E, Hamelin A, Stoicoviciu L (1987). J Electroanal Chem Interfacial Electrochem.

[22] Yasuda S, Kumagai R, Nakashima K, Murakoshi K (2015). J Phys Chem Lett.

[23] Bunge E, Nichols R J, Roelfs B, Meyer H, Baumgärtel H (1996). Langmuir.

[24] Iqbal S, Bondü C, Baltruschat H (2015). J Phys Chem C.

[25] Stegemann B, Bernhardt T M, Kaiser B, Rademann K (2002). Surf Sci.

[26] Jung G, Park H, Rhee C K (1998). J Electroanal Chem.

[27] Wang R Y, Wessells C D, Huggins R A, Cui Y (2013). Nano Lett.

[28] Bard A A, Faulkner L R (2001). Electrochemical Methods.

[29] Graham A R, Kaiman S (1952). Am Mineral.

[30] Nayeb-Hashemi A A, Clark J B (1984). Bull Alloy Phase Diagrams.

