# Supplementary material for: Antimony deposition onto Au(111) and insertion of Mg
Source: Beilstein J Nanotechnol. 2019 Dec 18;10:2541–52. doi: 10.3762/bjnano.10.245 (PMC6941451; doi:10.3762/bjnano.10.245)
Supplement: File 1 — Additional experimental details. [file Beilstein_J_Nanotechnol-10-2541-s001.pdf]

BEILSTEIN JOURNAL OF NANOTECHNOLOGY

Supporting Information

for

Antimony deposition onto Au(111) and insertion of Mg

Lingxing Zan, Da Xing, Abdelaziz Ali Abd-El-Latif and Helmut Baltruschat

Beilstein J. Nanotechnol. **2019,** *10,* 2541–2552. doi:10.3762/bjnano.10.245

Additional experimental details

License and Terms: This is a supporting information file under the terms of the Creative Commons Attribution License (https://creativecommons.org/licenses/by/4.0). Please note that the reuse, redistribution and reproduction in particular requires that the authors and source are credited.
The license is subject to the *Beilstein Journal of Nanotechnology* terms and conditions: (https://www.beilstein-journals.org/bjnano)

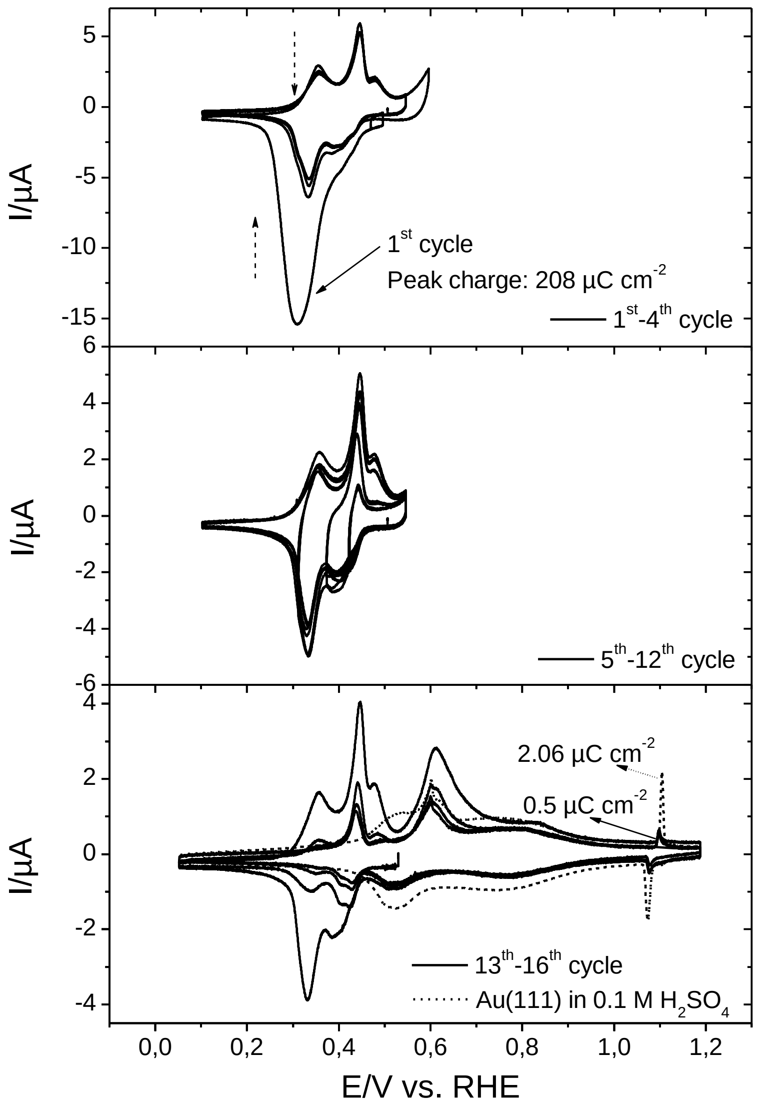

Figure S1: Cyclic voltammograms of Sb species/Au (111) in 0.1 M H_2SO_4 electrolyte saturated with Ar in H cell at the sweep rate of 10 mV s^{-1}. The Sb species/Au (111) was obtained after carrying out bulk deposition and dissolution of Sb on Au (111) in Sb containing electrolyte, as shown in Figure 2. The CV of fresh prepared Au (111) in 0.1 M H_2SO_4 electrolyte saturated with Ar in H cell at the sweep rate of 10 mV s^{-1} (bottom, the dot curve).

After carrying out the deposition and dissolution of Sb for several potential cycles in Sb containing electrolyte as seen in Figure 2, the potential was held at +0.5 V and the Au(111) was rinsed with plenty of 0.1 M H_2SO_4 electrolyte and transferred into 0.1 M H_2SO_4 electrolyte. The corresponding cyclic voltammograms were recorded and are shown in Figure S2. A large cathodic peak with a weak shoulder was observed at +0.3 V in the first potential cycle, and then it was separated obviously into two peaks which decreased with the increase of the cycle number. This means that the process of deposition/dissolution of Sb on Au(111) is irreversible and the deposited Sb species cannot be dissolved completely at the positive potential of +0.5 V

in Sb containing electrolyte. However, with the continuous potential scan in the blank electrolyte, it was indicated by sulphate adsorption/desorption spike at ~1.1 V that most of the adsorbed Sb species can be dissolved and the Au(111) surface reappeared again due to the rearrangement of surface Au atoms. The decrease in the charge of the spike at ~1.1 V is probably due to the increase in the roughness of the surface. Two cathodic peaks C1 and C2 appeared after the first potential cycle and they are probably attributed to the reduction of preadsorbed Sb(III) species and Sb(III) species from the electrolyte, respectively.

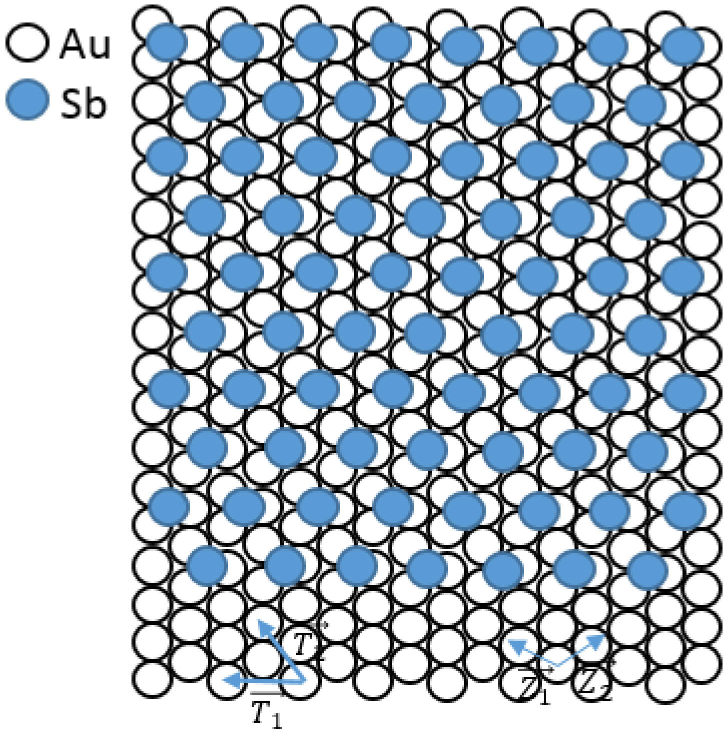

Figure S2: The adlayer $\left(\sqrt{3}\times\sqrt{3}\right)$ structure of Sb on the substrate Au(111) as a model. The two lattice vectors $\overrightarrow{Z_1}$ and $\overrightarrow{Z_2}$ are defined for the substrate (Au(111)) as a model. White ball: Au atoms; blue balls: Sb atoms.

.

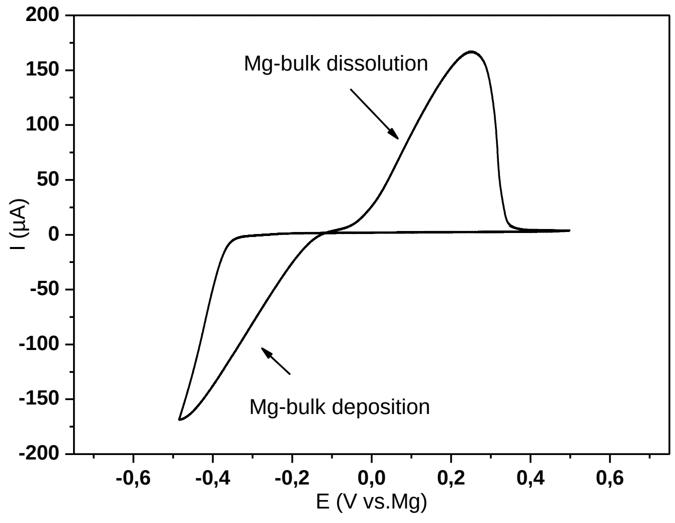

Figure S3: Cyclic voltammograms of Mg deposition/dissolution at single Au-electrode (Au 111) in MACC/tetraglyme at the sweep rate of 1 mV s^{-1}. Here are no peaks for the Mg-insertion and for the Mg-deinsertion.

Figure S2 shows the electrochemical deposition and stripping behavior of Mg at Au(111) (without Sb on the surface) electrode in 0.5 M $MgCl_2$ + 0.5 M $AlCl_3$ in tetraglyme. Bulk deposition of Mg starts at -0.33 V, which is around 0.1 V more overpotential than the bulk deposition on the Sb-modified Au(111) at -0.2 V. During the anodic going sweep, the current is still negative in the potential range of -0.5 V to -0.1 V due to the continuous deposition of Mg. At E > (-0.1 V) in the cathodic sweep, Mg start to be dissolved. The coulombic efficiency for the Mg-dissolution/deposition is about 95%. Compared with the CV in Figure 10, here are no peak for the Mg-insertion into Sb in the cathodic sweep and no peak for the Mg-deinsertion in the anodic going sweep, which are observed at 0.1 V vs Mg and at 0.25 V vs Mg in Figure 10 (The peak for the Mg-deinsertion starts already at 0.25 V vs Mg in Figure 10b).
